# 

**Published:** 1879-04-20

**Authors:** 


### TABLE OF CONTENTS.

ORIGINAL AND SELECTED ARTICLES.

Report of Some Cases from the Eye and Ear and Throat Clinic, by Prof. A. W. Calhoun, 121. Injuries of the Head—Bloodletting in Extravasation, by E. F. Wells, M. D., 123. Viburnum Prunifolium, byG. S. Glazener, M. D., 125. A Case of Leprosy—Elephantiasis Græcorum, 126. Malnutrition and Diarrhœa in an Infant, from Improper Feeding, by Dr. Jacobi, 128. The Bedford Alum and Iron Spring, by Prof. J. J. Moorman, M. D., 129. *Society Reports*—Medico-Chirurgical Association, 131.

ABSTRACTS AND GLEANINGS.

Post-Mortem Delivery, 137. Remedies for Dilating the Cervix During and Preparatory to Labor; The Treatment of Alcoholism, 138. Pathology of Hysterical Cough, 139. Arrest of Milk Abscess, 140. Prussian Blue in Malarial Fever; A Simple Aspirator, 141. Hypodermic Injection of Brandy, 142. The Plague; Marriage and the Microscope, 143. Rheumatism, 144. The Treatment of Acute Rheumatism; Fistula in Ano; Hyoscyamine, 145. Berlin Treatment of Scarlet Fever; Fatal Tetanus Following Ligation of Hemorrhoids; The Croton Oil Treatment of Nævus; Glycerine as an Enema in Constipation, 146. Is Urticaria Caused by Sulphate of Cinchonidia; Sulphuric Ether in Lumbago; The Topical Application of Chloral Medicated with Camphor; Battey's Operation, 147. Use of Chloroform in Diseases of the Heart; Warm Fomentations to the Head in Cases of Uterine Hemorrhage; Novel Use for Apomorphia; Herpes Zoster, 148. Epilepsy; Purulent Ophthalmia of Infants; Treatment of enlarged Glands in Scarlet Fever; Diphtheria, 149. Treatment of Erysipelas by Injections of Carbolic Acid; Occurrence of Herpes During the Administration of Arsenic; Treatment of Mammary Abscess; Obstinate Singultus Cured by Muriate of Pilocarpine; Paracotoin as a Remedy in Epidemic Cholera, 150.

SCIENTIFIC ITEMS.

Pregnancy in the Elephant; Experiment to Illustrate Geographical Processes, 151. A Hairy Water Tortoise; Caloptine; Absorption of Carbonic Oxide by Living Organisms; Meteorology for 1878; Self-Luminous Clocks; Disappearance of Nebulæ; Invisible Ink, 152.

PRACTICAL NOTES AND FORMULÆ.

Habitual Constipation; Cinchonia Alkaloid, 153. Diphtheria; Treatment of Burns; Poisoning by Rhus Toxicodendron, 154. Aconite; Oxalate of Cerium in Vomiting of Pregnancy, 155. Convulsions; Dog-fennel; Boracic Acid Ointment; Trichinæ in the Flesh of Geese, 156.

EDITORIAL AND MISCELLANEOUS.

American Medical Association; Medical College Association, 157. Southern Medical College; Importance of Medical Journals, 158. State Medical Societies; Locals; Our Advertising Department, 159. The Association of Medical Editors; Special Notices, 160.

